# Development and evolution of the metazoan heart

**DOI:** 10.1002/dvdy.45

**Published:** 2019-05-20

**Authors:** Robert E. Poelmann, Adriana C. Gittenberger‐de Groot

**Affiliations:** ^1^ Institute of Biology, Department of Animal Sciences and Health Leiden University Leiden The Netherlands; ^2^ Department of Cardiology Leiden University Medical Center Leiden The Netherlands

**Keywords:** arthropods, cardio genesis, chordates, endocardial cushions, endothermy, evolution, gene regulatory network, gene regulatory tool kits, mollusks, outflow tract, septation

## Abstract

The mechanisms of the evolution and development of the heart in metazoans are highlighted, starting with the evolutionary origin of the contractile cell, supposedly the precursor of cardiomyocytes. The last eukaryotic common ancestor is likely a combination of several cellular organisms containing their specific metabolic pathways and genetic signaling networks. During evolution, these tool kits diversified. Shared parts of these conserved tool kits act in the development and functioning of pumping hearts and open or closed circulations in such diverse species as arthropods, mollusks, and chordates. The genetic tool kits became more complex by gene duplications, addition of epigenetic modifications, influence of environmental factors, incorporation of viral genomes, cardiac changes necessitated by air‐breathing, and many others. We evaluate mechanisms involved in mollusks in the formation of three separate hearts and in arthropods in the formation of a tubular heart. A tubular heart is also present in embryonic stages of chordates, providing the septated four‐chambered heart, in birds and mammals passing through stages with first and second heart fields. The four‐chambered heart permits the formation of high‐pressure systemic and low‐pressure pulmonary circulation in birds and mammals, allowing for high metabolic rates and maintenance of body temperature. Crocodiles also have a (nearly) separated circulation, but their resting temperature conforms with the environment. We argue that endothermic ancestors lost the capacity to elevate their body temperature during evolution, resulting in ectothermic modern crocodilians. Finally, a clinically relevant paragraph reviews the occurrence of congenital cardiac malformations in humans as derailments of signaling pathways during embryonic development.

## Key Findings

- The cardiac regulatory toolkit contains many factors including epigenetic, genetic, viral, hemodynamic, and environmental factors, but also transcriptional activators, repressors, duplicated genes, redundancies and dose‐dependancies.
- Numerous toolkits regulate mechanisms including cell‐cell interactions, EMT, mitosis patterns, cell migration and differentiation and left/right sidedness involved in the development of endocardial cushions, looping, septum complexes, pharyngeal arch arteries, chamber and valve formation and conduction system.
- Evolutionary development of the yolk sac circulation likely preceded the advent of endothermy in amniotes.
- Parallel evolutionary traits regulate the development of contractile pumps in various taxa often in conjunction with the gut, lungs and excretory organs.

## 1 INTRODUCTION

In this review, we highlight the mechanisms of cardiac development in several taxa, starting with the evolutionary origin of the contractile cell as a proxy for the cardiomyocyte. It has been made credible that the last eukaryotic common ancestor (more than 1 billion years ago) has been an amalgam of several cellular organisms containing their specific metabolic pathways and signaling tool kits. During evolution, these tool kits diversified as the species diversified being part of the Cambrian explosion. Shared parts of these tool kits led to conserved elements acting in the development and functioning of pumping hearts and open or closed circulations in such diverse species as arthropods (>1 million extant species), mollusks (about 200 000 species), and chordates (an estimated 50 000 species). The genetic tool kits became more complex by gene duplications, the addition of epigenetic modifications, the influence of environmental factors, the incorporation of viral genomes, the cardiac changes necessitated by air‐breathing, and many others. Here, we evaluate mechanisms involved in cephalopods (as an example of mollusks) in the formation of three separate hearts, and in adult insects (an example of arthropods) in the formation of a tubular heart. A tubular heart is also present in embryonic stages of chordates, leading here to the septated four‐chambered heart, passing through stages with a first heart field (FHF) and a second heart field (SHF) in birds and mammals. The four‐chambered heart permits the formation of a high‐pressure systemic and a low‐pressure pulmonary circulation in birds and mammals, allowing for high metabolic rates and maintenance of body temperature. Crocodilians also have a (nearly) separated circulation, but their resting temperature conforms with the environment. Using data from cardiac embryology, we argue that endothermic ancestors lost the capacity to elevate their body temperature during evolution, resulting in ectothermic modern crocodilians. Finally, a clinically relevant paragraph (13) reviews the occurrence of congenital cardiac malformations in humans^1^ as derailments of signaling pathways during embryonic development.

## 2 EVOLUTION OF THE CONTRACTILE CELL

This early contractile cell was probably an endosymbiotic cell, an aggregation containing absorbed bacteria forming a nucleus, an enslaved α‐proteobacterium type mitochondrion, a prokaryote cilium, and others, exploiting actins, myosins, and tubulins as early as ~1 billion to 1.9 billion years ago.^2^, ^3^ These cells could migrate and contract and contacted each other by junctions and junctional complexes. Bilaterians evolved, forming a mesoblast, the anlage, to form many organs. The first one with a pulsatile, heartlike structure might have been akin to the Ediacaran *Kimberella*, about 600 million years ago and at the roots of the Cambrian explosion.^4^, ^5^ Several fairly different cardiac themes evolved with varying renditions of peristaltic and rhythmically beating hearts, driving extinct species and still thriving in extant Protostomia and Deuterostomia. We lack evidence about the cause of (mass) extinctions of many beautiful species, with the possible exception of the impact of one particular meteorite and the most recent impact of human influence. It is also likely that improperly adapted cardiac functions culled these inevitable evolutionary outcomes, as exemplified by lethal congenital cardiovascular malformations in current clinical practice. It has been postulated that a large number of prenatal deaths in mutant mice is related to malfunctioning of the cardiovascular system.^6^, ^7^ Here, the study of embryonic stages becomes necessary, not as a recapitulation of evolution but as a way of providing independently living larval and adult organisms that are adapted to their environmental challenges. In the meantime, the successful embryos need to stay alive through all their phases of growth and remodeling by recombination and diversification of available and newly recruited elements of their (signaling) tool kits as used by Erwin.^5^

## 3 CARDIAC PRECURSORS

It is tempting to consider hearts of different chordate species as points on a linear arrangement. This is an oversimplification, as extant species have developed independently from (common) ancestors. This holds true even more for embryonic stages as intermediates to the adult forms. Human and mouse heart development, as well as chicken and Zebrafish, have been studied most extensively, usually in combination with explanations on (molecular) mechanisms related to congenital cardiac malformations. Mechanisms described in various vertebrates have evolved over different evolutionary time spans, as fish and amphibians evolved early. The first mammals separated from reptilian ancestors ~350 million years ago, whereas birds came much later in existence (~150 million years). This resulted in an evolution of varying and increasing complexities. The astonishing success of the identification of similar molecular key elements underlying cardiac development in diverse invertebrate and chordate species^8^ has dominated the idea of common conserved homologies. However, new elements including epigenetic regulation and hemodynamic forces have been added during the millions of years of evolution, needing a new synthesis of concepts of peristaltic and beating pumps in open and closed circulations.

**Figure 1:**
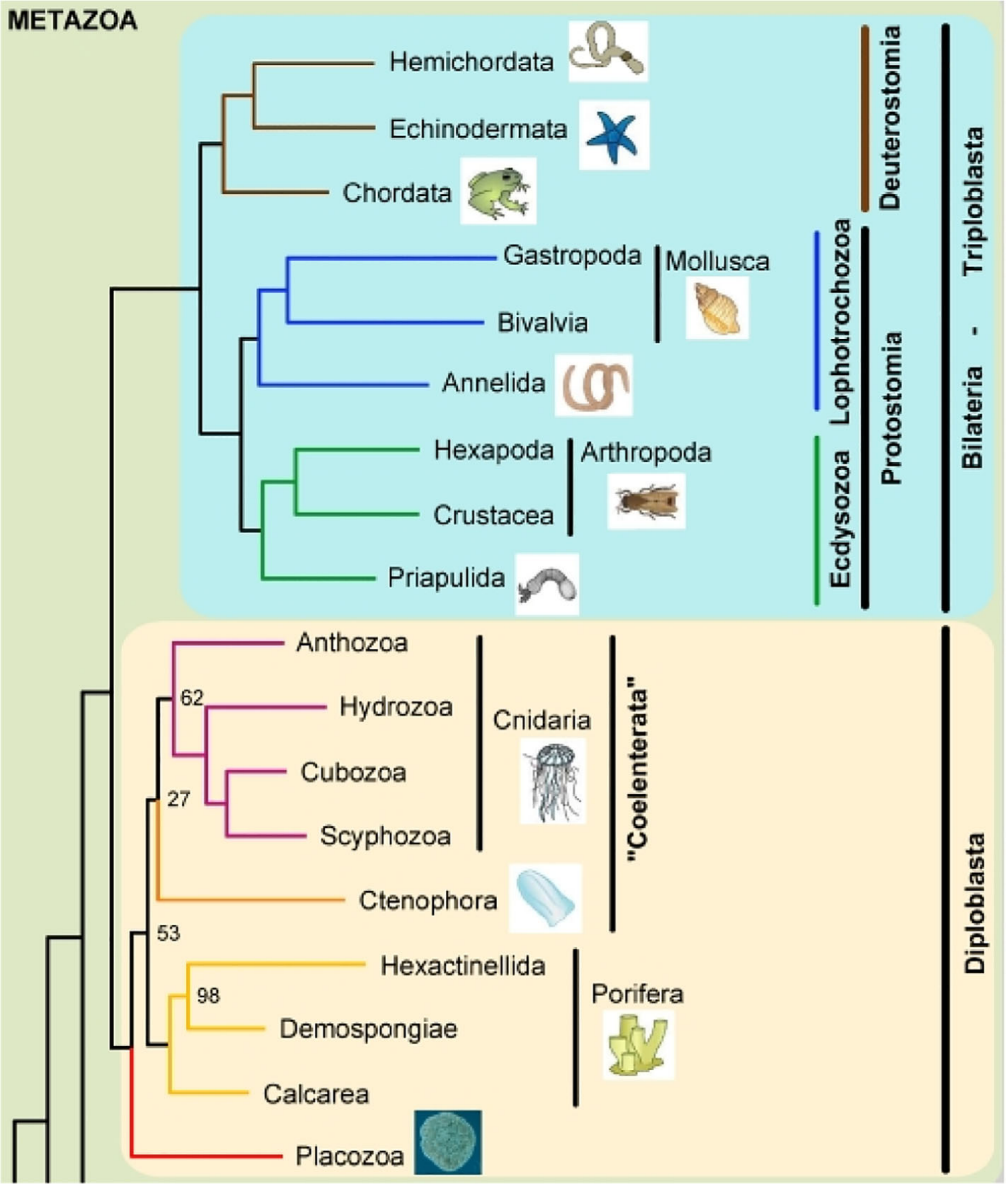
Phylogenetic relationships to demonstrate the position of arthropods, mollusks, and chordates among the bilaterians and metazoan (After^9^)

It is evident that contracting muscular tubes have found their ways in very divergent taxa such as protostomes (ecdysozoans: arthropods, including insects; lophotrochozoans: annelids, mollusks, including octopus), and deuterostomes (echinoderms, chordates, including vertebrates). Figure 1 (after Schierwater^9^) shows the phylogenetic relationships of these taxa. Their tubes perform analogous functions but are anatomically nonhomologous, supporting the concept of convergent evolution (homoplasy) even to the extent that a three‐chambered heart is present in cephalopods (a systemic heart comparable with a vertebrate left ventricle, and two respiratory branchial hearts in octopus) and in most reptiles, as in turtles. Furthermore, supporting wing hearts (insects), accessory hearts (hagfish), and muscularized lymph hearts (amphibians) are present.

**Figure 2:**
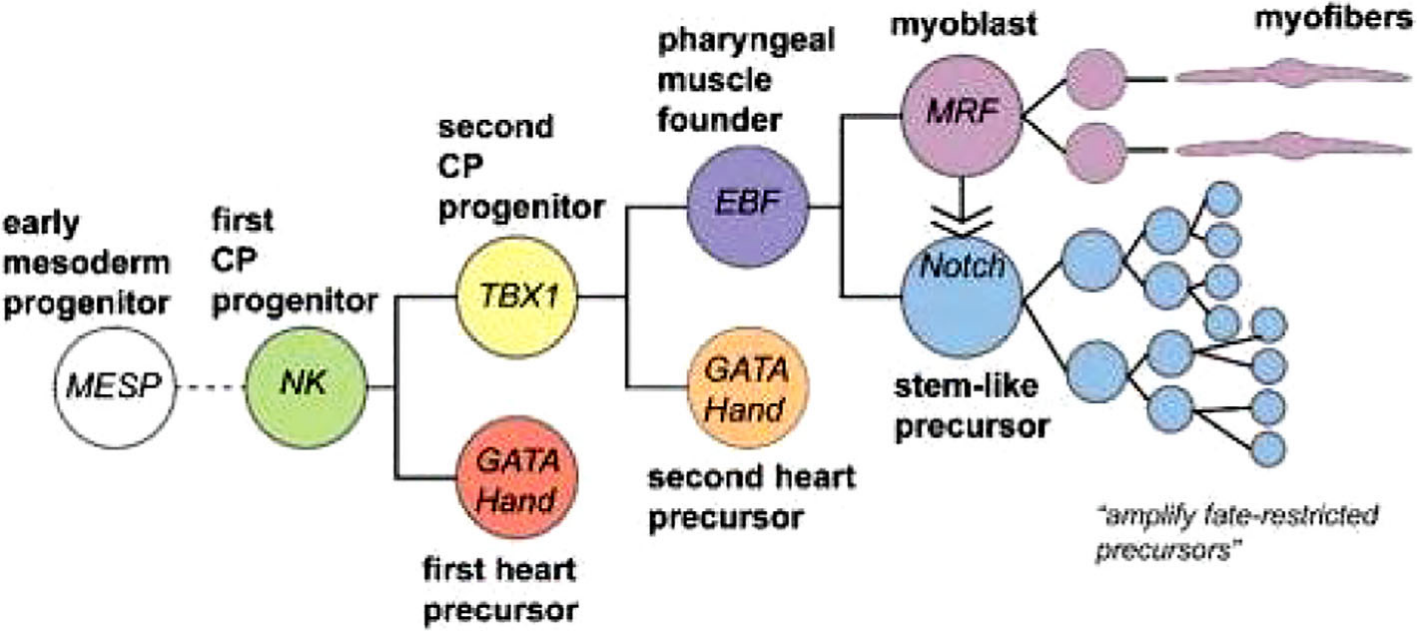
Cardiopharyngeal ontogenetic motif in *Ciona*. In ascidian embryos, cell fates are first restricted to a few progenitors, which are secondarily amplified (From Anderson and Christiaen^59^)

In tunicates (such as the ascidian *Ciona*), heart development has been studied extensively^10^, ^11^, ^12^ and compared to the vertebrate sister group. Ascidians possess a general chordate body plan including notochord, central nervous system, lateral muscles, and a ventral endodermal pharynx with gill slits. The heart beats peristaltically, although potentially in a reversible fashion through an open circulatory system, comparable to that of insects,^13^ and is not lined by endothelial cells. It functions only after a clade‐specific morphogenesis,^11^ in which it transforms from a free‐swimming larva into a sessile filter‐feeding adult. The heart rudiment develops from a single pair of blastomeres in the 64‐cell stage, together with several other body muscles.^10^, ^14^ The descendants of these blastomeres express *Mesp*, which has an essential role in the speed of migration and the specification of multipotent cardiac progenitor cells.^15^ In ascidians, *Mesp1/2* is regulated by beta‐catenin, *Tbx6*, and others. *Mesp1*, likely representing the first element of the cardiac tool kit (Figure 2), probably functions as a dual regulator, activating *Ets1/2* and *Hand‐like* and repressing *Raldh2*.^11^ Excess cardiac tissue by Mesp‐driven *Ets1/2* overexpression may lead to a two‐compartment phenotype,^16^ although being distinct from a cardia bifida as observed after retinoic acid treatment of chicken embryos.^17^ In chicken embryos, GATA 4/5/6 is expressed in both precardiac mesoderm and gut epithelium.^18^ In mouse embryonic stem (ES) cells, *Mesp* activates *Hand2, Nkx2.5*, and *GATA4*, while repressing genes involved in the maintenance of multipotency.^19^ In a human ES cell line, performing a whole genome‐based transcriptome study, the Mesp1 regulatory network showed a highly dynamic up‐regulation of extracellular matrix proteins.^20^ A survey of 647 patients with congenital outflow tract (OFT)‐related heart defects delivered seven patients with Mesp1 mutations,^21^ suggestive of its contribution to the development of congenital heart disease. The differentiation of the precardiac mesoderm in an evolutionary context is driven by interactions between a restricted set of genes. A handful of orthologues (*Nkx2.5/Tinman, GATA 4/5/6/Pannier, Hand1/2, Tbx5, Mef2c*) in mesodermal precardiac tissues are referred to as “heart regulatory kernel”^11^, ^22^, ^23^ or “core cardiac transcription factors,“^24^ followed by various sets of “terminal selectors”^25^ or “patterning genes,” 24 with very different outcomes in ascidians and vertebrates,^26^ which can be extended to other taxa such as insects and mollusks.^27^ In arthropods, the various types of circulation have recently been described.^28^ The *Drosophila* open circulatory system^29^ contains a heart consisting of 52 cell pairs surrounding a cardiac cavity and lined by nonmuscle pericardial cells with shared ontogenetic origin. Notwithstanding different morphogenetics, vertebrate and fly heart development show many commonalities. In *Drosophila tailup (Islet1* homolog), among others also essential for the formation of the alary muscles connecting the heart tube to the exoskeleton, is part of a cardiac regulatory unit that includes *tinman* (the fly homolog of Nkx2.5), *pannier* (GATA homolog), *decapentaplegic* (BMP family member), and *wingless* (Wnt homolog), and all can be included in the ancestral core set of cardiac transcription factors. These transcription factors have a dynamic expression pattern during heart development, suggesting that their function can vary depending on timing and cellular context.^27^, ^30^ In chicken, heterotopic transplantation of the organizer Hensen's node is able to induce ectopic cardiac differentiation in the host, expressing early cardiac markers, including Nkx2.5, but also involving fibroblast growth factor (FGF) and transforming growth factor (TGFβ) signaling.^31^

In mollusks, essential functions are performed in determining left/right asymmetry and chirality by the already described cardiac signaling network, such as decapentaplegic/BMP, Nodal signaling, and also dose‐dependent formin genes.^32^ Due to the complex evolution and diversification of these species, various interactions are proposed.^33^ Nodal signaling is a key factor in the determination of left/right asymmetry, characteristic for body plan development in, for example, snails,^34^ and also in cardiogenesis.^35^ In the freshwater snail *Lymnaea*, a set of tool kit genes involved in vertebrates both in cardiogenesis and in left/right body asymmetry (*Nodal, BMP4, FGF8, Lrd, Inversin, Brachyury*) is expressed in embryonic stages^36^ and related to the sidedness in other molluscan species.^37^ The determination of chirality in snails is complicated by a system of delayed inheritance in which the maternal genotype determines the phenotype in the offspring.^38^ Heart development in the more elaborate cephalopods has been studied in more detail, showing a closed endothelium‐lined circulatory system. This probably developed in parallel to the vertebrate system as in general invertebrates, including other mollusks present with an open vascular circulation that changes considerably, establishing first a larval heart and later in development a true heart.^39^ In squid, vascular endothelial growth factor (VEGF) and FGF receptors have been found in embryonic vessels,^40^ pointing to conserved signaling pathways; however, more studies are necessary to establish the gene regulatory networks in molluscan cardiogenesis. The circulation system in hagfish, the most ancestral of extant vertebrates, is special as it includes several accessory pumps to the branchial heart, which is equivalent to the heart in other vertebrates.^41^ These hearts exhibit limited looping and absent OFT elements.

**Figure 3:**
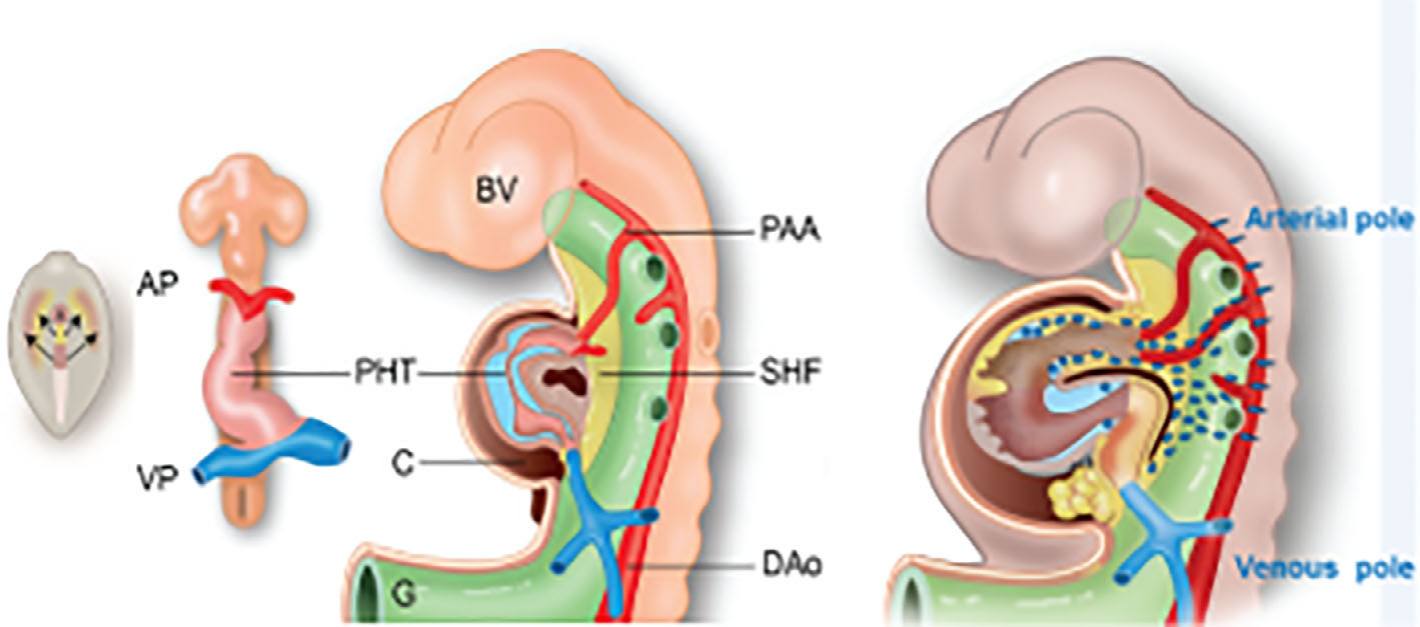
Development of the heart tube. Left: The cardiac crescent contains both FHF and SHF precursors (arrows). Center left: The slightly looped primary heart tube connects the venous and arterial poles. Center right: In a later stage, the still undivided heart with an atrial component with the cardinal veins and the pulmonary vein. The ventricular compartment shows anterior (future right) and posterior (future left) sides. The cardiac jelly has developed into two cushion complexes: the atrioventricular and outflow tract cushions (blue). The arterial pole shows the aortic sac and with the attached pharyngeal arch arteries. The dorsal mesocardium starts to disrupt (brown hole). The second heart field is depicted in yellow. Right: The derivatives of the anterior and posterior parts of the second heart field (yellow) together with the migrated neural crest cells (blue‐green). AP, arterial pole; AV, atrioventricular; Dao, dorsal aorta; FHF, first heart field; OFT, outflow tract; PAA, pharyngeal arch arteries; PHT, primary heart tube; SHF, second heart field; VP, venous pole

In chordates, the development of the heart is a multistep process involving the lateral plate mesoderm‐derived first heart field (FHF) and second heart field (SHF), although it is argued that both form a continuum in time and space. In principle, the FHF forms the left ventricle while the SHF adds cells to the venous and arterial poles, contributing to the atria and right ventricle, respectively. The details will be discussed in the next paragraphs (see also Figure 3).

## 4 THE FIRST HEART FIELD

The primitive cardiac tube has been considered as the sole primordium of all cardiac segments^42^ until the detection of the two heart fields in chicken,^43^, ^44^ in mouse,^45^, ^46^ and in Zebrafish.^47^ The molecularly distinct precursors of the heart fields both express *Mesp1*, showing that FHF cells are unipotent, but SHF precursors are bipotential.^48^ It became clear that the primitive cardiac tube (Figure 3) derives from the FHF and basically has a left ventricular identity. This identity prominently displays Tbx5 and Nkx2.5 expression in the mouse embryo.^49^ In the embryo, earlier cardiac crescent progenitors of the SHF are positioned medially to the FHF (Figure 3 Left). Embryological studies indicate that the FHF‐derived left ventricle is ancestral,^24^, ^50^ with progenitor cells of the arterial and venous poles^48^, ^51^, ^52^, ^53^, ^54^ positioned posteriorly to the ventricular field. These will be guided by chamber‐specific gene interactions involving Tbx1, Tbx2, Nkx2.5, and ANF or will embrace a ventricular fate in the absence of retinoic acid signaling,^55^, ^56^, ^57^ which also belongs to the cardiac tool kit. Gene regulatory networks determining the interactions in the various precursors have been summarized by Herrmann et al^58^ and Anderson and Christiaen.^59^ See also Figure 2.

## 5 THE SECOND HEART FIELD

There is a necessity to increase cardiac recruitment in the broader field of the SHF^60^ before potentiating new compartments.^22^ The SHF cells (Figure 3 Center right, Right) have a delayed differentiation but are more proliferative.

The SHF provides initially for precursors for both the arterial and the venous poles.^53^, ^54^ A caudal proliferation center contributes to both the anterior and posterior poles of the heart.^61^ Later, the SHF separates in the anterior and posterior heart fields, accompanied by the breakthrough of the dorsal mesocardium (Figure 3 Center right). The anterior field provides for the right ventricle, the OFT, and part of the ventricular septum, but not the atria, epicardium, or coronary vessels, while the OFT smooth muscle cells are of mixed SHF and neural crest (NC) origin.^62^ The cardiac epicardium and part of the coronary arteries find their origin in the posterior SHF.^63^ Tbx5 and Tbx1, expressed in the SHF mesoderm, display different functions in the anterior and posterior parts^54^, ^57^ and, together with retinoic acid, regulate the segregation of arterial and venous pole progenitors. Heterospecific interactions between a small number of the transcription factors Tbx5 and Nkx2.5, and GATA4, also part of the cardiogenic tool kit, underlying gene expression patterns in specific tissues, have been demonstrated in mammalian cardiogenesis.^64^ However, this shows much complexity and requires fine‐tuning involving additional partners, including histone modifications, epigenetic regulation,^23^, ^65^, ^66^ involvement of hemodynamics,^67^, ^68^ regulation of cell movement,^69^ and participation of microRNAs^70^, ^71^ to provide for expansion of cardiac progenitors, compartments, and cell differentiation.

## 6 THE POSTERIOR SECOND HEART FIELD

The posterior SHF is interesting in amniotes as it shows the development of two organ systems, the lungs and the atria. The co‐evolution of lungs and the atrial compartment of the heart, including the atrial septum, is probably based on the conserved interaction of Tbx5‐Wnt2/2b signaling pathways,^72^ found in the posterior SHF of the lateral plate mesoderm in air‐breathing amphibians, as well as in amniotes. This makes the accidental appearance of symmetric atrial and lung development in congenital forms of atrial isomerism understandable. An important derivative of the SHF with respect to atrial development is the dorsal mesenchymal protrusion (DMP), involved in atrial septation by fusion with the endocardial atrioventricular (AV) cushions.^73^ The progenitors within the posterior SHF give rise to multipotent cardiopulmonary progenitors, a process that is regulated by hedgehog (Shh/Gli) signaling from the foregut endoderm.^74^ Other paracrine signaling pathways in amphibians and amniotes also involve retinoic acid, GATA4/5/6, and Bmp. It is interesting to note that a subpopulation of myocardial cells added to the venous pole does not express Nkx2.5 and is related to the development of the cardiac pace‐making and conduction systems.^75^, ^76^ Tbx5 as an essential transcription factor for heart development interacts with the transcriptional repression machinery of the nucleosomal remodeling complex,^77^ which evolved during the early diversification of vertebrates (excluding fish) together with the evolution of cardiac septation.^78^, ^79^, ^80^

## 7 THE ANTERIOR SECOND HEART FIELD

The SHF is active from fish onward,^81^, ^82^, ^83^ although with a diverse outcome as there are more than 25 000 species of fish described. In general, the anatomy of the fish heart has conveniently been divided in four main types mainly based on ventricular structure and the presence/position of coronary vessels.^83^ The heart is tubular with three compartments: the sinus venosus, the atrium, and the ventricle with sinoatrial (SA) and AV valves between the respective compartments. The OFT contains multiple valve leaflets. In sharks, several species‐dependent rows of conal valves are present as in the myocardial part (conus arteriosus) of the “ancient” teleost heart. In several species, smooth muscle cells and fibroblasts^84^ have been reported in the wall of the conus. In the “modern” teleost, a single valve set has been maintained, probably as a result of evolutionary loss. In lungfish, the muscular conus is free from valve leaflets, while a spiral valve is reported in the more distal truncus arteriosus, which is also myocardial. The smooth muscular elastin‐rich bulbus arteriosus is mostly considered as the vascular wall distal to the OFT valves and probably akin to the arterial trunk of land‐based vertebrates (reviewed by Grimes and Kirby^83^). In the early mammalian heart, the “bulbus cordis” usually identifies the myocardium, giving rise to the OFT. However, this includes the right ventricle as well as the conus and the truncus arteriosus, as a consequence giving rise to many misinterpretations.^85^ In reptiles, birds, and mammals, the conus is remodeled and incorporated into the right(−sided) ventricle, whereas in fish there is no right ventricle. In Zebrafish, two waves of SHF‐derived cells migrate to the arterial pole. Ventricular progenitors migrate directly to the arterial pole, whereas OFT progenitors become sequestered temporarily in the core mesoderm of the second pharyngeal arch, meanwhile down‐regulating Nkx2.5. Hereafter, they migrate and differentiate into OFT lineages residing in both conus and bulbus,^81^ with different sensitivities to *fgf8*. In embryonic Zebrafish, progenitors are continuously added to the ventricle, whereas late/differentiating progenitors are added to the bulbus, thereby lengthening the FHF‐derived primitive heart tube.^47^

In amniotes, the right ventricle is a new compartment derived from the anterior SHF, requiring canonical Wnt/β‐catenin signaling.^86^ It develops in concert with the evolutionary new addition of air‐breathing, requiring functioning lungs. In lungfish, this is heralded by partial septation of both the atrium^87^ and the ventricle,^88^ pointing toward separation of the pulmonary and systemic blood flows. In amphibians, only the atrium, but not the ventricle, is septated, but this group has acquired the potency of skin‐breathing, probably requiring another type of circulation. In amniotes, septation progressed by remodeling of the OFT, originally only serving as outflow of the primary heart tube. SHF‐derived NKx2.5‐positive myocardial precursors are asymmetrically contributing to the pulmonary and aortic sides of the OFT, resulting in a lengthening of the pulmonary trunk and a distal positioning of the pulmonary semilunar valve, also referred to as the “pulmonary push.”^89^ The OFT of the doubled ventricle is now doubly connected to the aortic sac compared to the single connection in the proposed ancestral vertebrate and in ascidians. Elongation of the cardiac tube takes place by progressive addition of SHF‐derived cells (reviewed by Cortes et al^90^), requiring *disheveled* and *Wnt5a*.^91^

Apart from SHF progenitors, the anterior (arterial) pole of the heart receives an additional population, the neural crest cells (NCCs).^92^, ^93^, ^94^, ^95^, ^96^ The interesting mix of SHF and NCC populations provides for several intricate remodeling processes, including myocardial differentiation (more specifically of OFT and right ventricle), OFT cushion morphogenesis, semilunar valve formation, separation of the aortic and pulmonary channels, and arterial vessel wall differentiation.

In ancient teleosts, such as *Arapaima gigas*, NCCs might be encountered in the bulbus segment of the heart as indicated by the presence of pigment cells.^97^ (See Reyes‐Moya et al.^98^) Lineage labeling in Zebrafish demonstrated that NCCs from the classical cardiac neural crest (caudal to the otic placode) could give rise to MF20‐positive muscle cells in the complete cardiac tube. In addition, a more rostral segment of the neural crest is also involved.^99^ Laser ablation of relevant parts of the neural crest resulted in severe cardiac malformations.^100^ In (transgenic) Zebrafish, NCCs are reported to migrate as two populations to the embryonic heart tube. The first wave passes through pharyngeal arch 1 and arch 2 and gives rise to about 10% of the cardiomyocytes over the entire tube. SHF‐derived cardiomyocytes mostly occupy the distal third part of the heart.^96^ The much later second wave of NCCs in Zebrafish^96^ migrates along the sixth pharyngeal arch, enveloping the endothelium of the ventral aorta and invading the bulbus arteriosus. FGF inhibition prevents contribution to the OFT but not the integration in the heart tube, while NCC ablation results in restricted recruitment of SHF cells. In mice, PO‐1‐marked NCCs were found as undifferentiated cells nested in the myocardium of the left ventricle, excluding the right ventricle. These cells have the potential to differentiate into cardiomyocytes after injury.^101^ It is interesting to note that in chicken, early and late nonmyocardial NCC migration waves were indicated populating the OFT septal complex and pharyngeal arches, respectively.^102^ The interactions in birds and mammals with their inherent OFT septation is further discussed below.

## 8 CARDIAC SEPTATION

**Figure 4:**
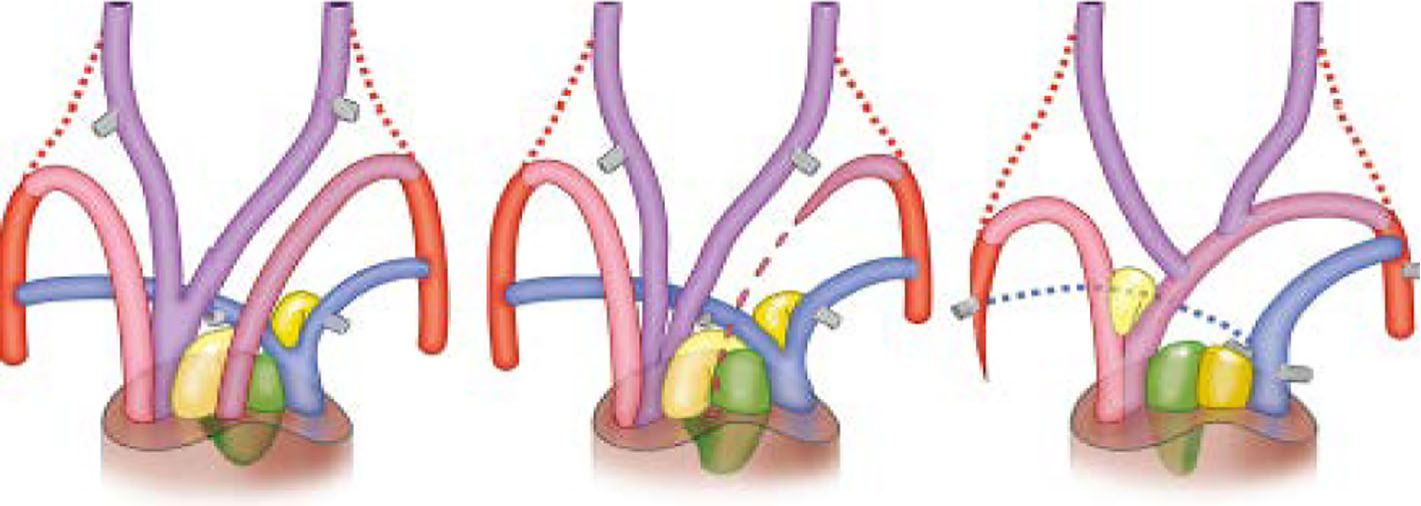
Outflow tract and pharyngeal arch arteries viewed from ventral in reptile (left), bird (center), and mammal (right). The carotid arch arteries (PAA3) are depicted in purple, the aortic arches (PAA4) in pink, the pulmonary arches (PAA6) in blue, and the dorsal aortae or rudiments in red. Note the disappearance of the left fourth PAA in birds (center) and of the right sixth PAA in mammals (C). As a consequence, the NC‐derived part of the outflow septum (green) associates with the SHF‐derived aortic flow divider (light yellow) in birds (B) or the SHF‐pulmonary flow divider (dark yellow) in mammals (D), resulting in two different OFT septa. NC, neural crest; OFT, outflow tract; PAA, pharyngeal arch arteries; SHF, second heart field (From Poelmann et al^104^)

It is evident that cardiac septation is restricted to several classes of vertebrates—lungfishes, amphibians, reptiles (including birds), and mammals—as it is related to the advent of air‐breathing and the accompanying presence of lungs requiring separation of pulmonary (low‐pressure) and systemic (high‐pressure) circulation. Complete septation, resulting in four chambers, is encountered only in adult crocodilians (for a critical comment, see “Evo‐devo aspects of congenital malformations paragraph 13”), birds, and mammals. Most amphibians, with the exception of lungless salamanders, present with a septated atrium only. The proximal part of the *Xenopus* single ventricle derives from the FHF, while the distal part feeding the OFT derives from both FHF and SHF,^103^ indicating an overlap of the cardiac fields. Furthermore, in contrast to birds and mammals, it is only the SHF and not the NCC that forms the incomplete spiral septum of the amphibian OFT.^103^ In reptiles, birds, and mammals, the composition of the OFT septation complex presents a combined participation of NCCs and SHF^104^ (Figure 4). The majority of reptiles (lizards, snakes, turtles) have an anatomically partially divided ventricle.^80^, ^105^ Varanid lizards and pythons have a functionally septated ventricular compartment during systole,^106^ reducing shunting between systemic and pulmonary blood.

**Figure 5:**
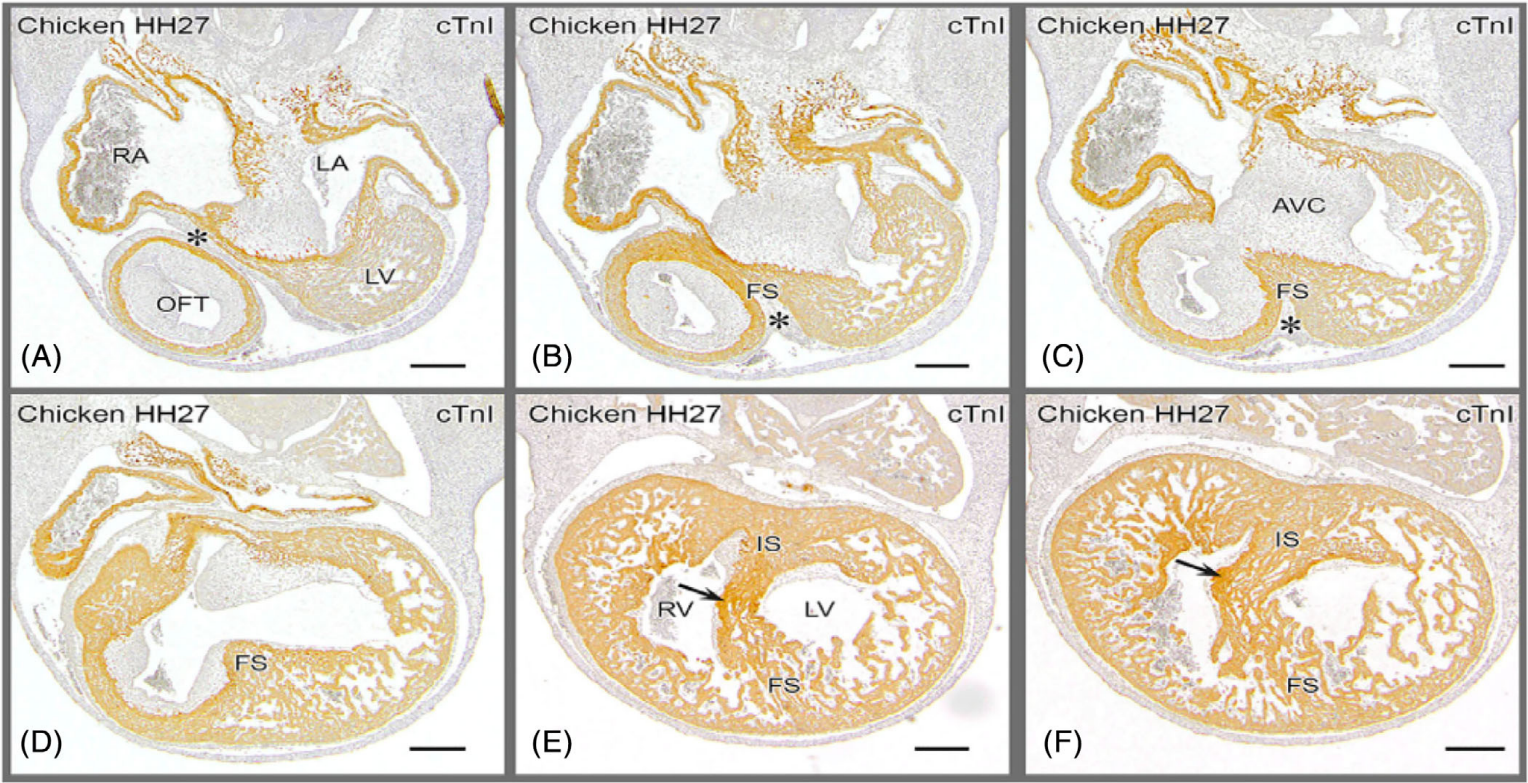
Ventricular septation in a chicken embryo. Merging of folding and inlet components before septation is finished. A: Most cranial, showing the epicardial cushion (*) between the outflow tract and the right and left atria, with the AV cushions in between. The left ventricle is grazed in the section. B,C: Cranial level of the folding septum between the OFT and the fused atrioventricular cushions. D: Folding septum borders the interventricular foramen. **E:** AV cushions attached to the flanks of the IS, where the tip of the septal OFT cushion is found (arrow). IS and FS have fused and constitute the floor of the interventricular foramen. E,F: Right AV junction is present in the right ventricle immediately above the arrow and can be traced downstream in D. AV, atrioventricular; AVC; atrioventricular cushions; FS, folding septum; IS, inlet septum; LA, left atria; LV, left ventricle; OFT, outflow tract; RA, right atria; RV, right ventricle (from Poelmann et al^80^)

During embryonic development, hearts of all vertebrates exhibit a kind of looping, which is governed by a right‐handed signaling pathway involving Nodal, BMP, and Pitx2.^107^ In fish, addition of SHF‐derived cells^47^ results in a limited elongation of the primitive heart tube, probably accompanied by a restricted repositioning of atrium and ventricle. In other vertebrates, the elongation process continues, probably by more massive addition of SHF cells to the FHF‐derived primitive heart tube. At the venous pole, this gives rise to the atrium. Septation of the atrium is most likely a phenomenon invoked by formation of the lungs, as will be related in a following paragraph. At the arterial pole, the SHF provides for the OFT that is serially connected to one ventricle. In mammals this will become the right ventricle, and in non‐crocodilian reptiles probably the cavum pulmonale (see below). Formation of the complete septum between left and right ventricles (Figure 5), however, is very complicated, indicated by the high percentage of ventricular septal defects (VSDs) in human congenital heart disease (reviewed in Gittenberger‐de Groot et al^108^). In non‐crocodilian reptiles, usually several septa are present between three interconnected compartments, being the cavum pulmonale, cavum venosum, and cavum arteriosum. The homology and origin of these cava from FHF or SHF are uncertain. The cavum arteriosum resembles mostly the mammalian left ventricle in position as it feeds the systemic aorta (in reptilians, the right fourth pharyngeal arch artery). The cavum pulmonale may resemble the mammalian right ventricle as it feeds the pulmonary trunk, but also the visceral aorta (in reptilians the left fourth pharyngeal arch artery). The cavum venosum is usually a smaller compartment located intermediate between the atria, the other ventricular cava, and the OFT. Functionally, the mixing of blood from the different cava is probably a minor issue considering the myocardial architecture of parallel trabeculations^109^, ^110^, ^111^ and the fluid characteristics of blood.^112^ The myocardial elements separating these chambers have been given various functions and names over the last century. The definition depended on their position in the body (horizontal and vertical septum^113^), their place in the heart (Bulbo‐auricular Sporn, Bulbusleiste^114^), bulbus lamella, muscular ridge, aorticopulmonary septum (reviewed in Jensen et al^115^), or their appearance during embryonic development (folding septum, inflow septum, OFT septal complex^67^, ^80^). The participation of the FHF and SHF with these structures has not been established, making the determination of homology with the septal structures in mammals hazardous. It is tempting to nominate the mammalian interventricular septum as the border between left and right ventricles based on the expression on FHF‐ and SHF‐related patterns; however, the evolution and development of the interventricular septum with its inlet (probably FHF‐derived) and folding (probably both FHF‐ and SHF‐derived) constituents is too complicated for this simplification.^79^, ^80^, ^116^ Further investigations determining the genetic networks in the septal complexes of reptiles, birds, and mammals are warranted before a more definitive conclusion can be drawn concerning the homology of the cava and ventricles and their separating myocardial and mesenchymal elements.

## 9 SEMILUNAR VALVES AND THE OFT

The usual three‐leaflet semilunar valve configuration in mammals and birds consists of two sets: one in the aorta and one in the pulmonary trunk.^117^ As a congenital malformation in humans, bicuspid valves are quite common (see also “Evo‐devo aspects of congenital malformations”). Reptiles nearly always present with bicuspid semilunar valves in all three major arteries, two persisting aortas. and one pulmonary trunk.^118^ In fish with one undivided OFT, the conal/truncal valves have many phenotypes, including multiple rows (elasmobranchs), a single valve (teleosts), and a spiral truncal valve (lungfish), as described above. The area in which the valves are embedded is very dynamic during embryonic development. It consists of epicardial, myocardial, endocardial, endothelial, and mesenchymal (smooth muscle and fibroblast) specializations from the SHF, supplemented by NC‐derived mesenchymal, smooth muscle, and neuronal cells and fibroblasts. The interactions between these cell populations in the OFT dictate the outcome of the different phenotypes.

The delineation of the OFT and its components has received much attention and discussion in comparative cardiac embryology.^83^, ^119^, ^120^, ^121^, ^122^, ^123^ This has rendered sometimes conflicting hypotheses about compartmental properties and homologies in comparative vertebrate studies and the description of congenital malformations in the human population, with consequences for more recent aspects of gene regulatory networks and protein interactions. It seems evident that the OFT in fish, for example, consists of a proximal myocardial conus arteriosus as well as the more distal bulbus arteriosus rich in smooth muscle cells as well as elastin. The evolutionary origin of the latter is still uncertain, although both conus and bulbus are SHF‐derived^123^ with an additional contribution of NCCs. Due to heavy remodeling in mammals, the distinction in conus and bulbus is far from clear. Our approach is a three‐part distinction, from proximal to distal: (1) the myocardial tube harboring the proximal endocardial OFT cushions and involved in ventricular septation; (2) the transitional zone with distal endocardial cushions developing into the arterial roots with the developing semilunar valves; and (3) the arterial walls of the pulmonary trunk and the ascending aorta. This distinction essentially mirrors the description by Grimes et al.^85^

## 10 THE OFT ENDOCARDIAL CUSHIONS

The endocardial cushions serve a double function in cardiogenesis: formation of the AV and semilunar valves, and separation of the systemic and pulmonary flows (Figure 5).

Endocardial cushions, beginning as acellular cardiac jelly, will develop in the AV canal as well as in the OFT. The cardiac jelly, sometimes considered the myocardial basement membrane, contains extracellular matrix components such as hyaluronan and aggrecan^124^ and will become cellularized by endocardial‐mesenchymal transition (EMT) primarily from the overlying endocardium.^125^, ^126^, ^127^, ^128^ Secondarily, other cell populations take part in expanding the cushions. These include epicardial cells migrating from the AV junction in the case of AV cushions,^129^, ^130^, ^131^ and epicardial cells and NCCs in the case of OFT cushions.^103^, ^132^ Each individual cushion probably receives its own balanced set of mesenchymal cells.^130^, ^131^, ^133^ In chicken embryos, it is shown that the formation of the semilunar valves highly depends on the interaction of endocardial and NC‐derived mesenchymal cells guided by hemodynamical forces sculpting the OFT.^67^ It is noteworthy that also in these events of cardiogenesis, asymmetric development is apparent, although the main leaflets of the aortic and pulmonary semilunar valves originate from the same two OFT endocardial cushions. Only the so‐called non‐facing leaflets^117^, ^133^ may have a different contribution of NCCs. Indeed, aortic bicuspidy in humans is far more common than pulmonary bicuspidy, as is also supported by studying a hamster strain.^134^ Another asymmetric event is the expansion of the pulmonary trunk compared to the aorta, also called the pulmonary push,^89^ which is probably responsible for the more distal location of the pulmonary orifice and valve compared to the aortic orifice. The expansion of the pulmonary trunk also corroborates the absence of rotation of the OFT, as has been advocated.^135^, ^136^, ^137^

EMT of the endocardial area is regulated by TGFβRIII, Cadherin 11, and Postn encoding for the extracellular matrix protein periostin.^138^ Gene regulatory networks in the cardiac tool kits have been studied in mammalian embryos. These include but are not limited to Notch1,^139^ Slit‐Robo,^140^ Krox 20,^141^ Wnt signaling,^142^ TGFβ signaling,^143^ NFATc1,^144^ ADAM17,^145^ Msx,^128^ and matrix proteins such as laminin,^146^ versican,^147^, ^148^ periostin,^149^ hyaluronan,^145^ tenascin and fibrillin,^68^ elastin, collagens, and others (see Pucéat^150^).

The other functions of the endocardial cushions are involvement in both AV septation^151^ and OFT separation.^152^ In the OFT, an associated role of NCCs is obvious, although the exact interaction with SHF mesenchymal cells is far from clear. This is partly due to the dynamic evolutionary history of this area. In fish and amphibians, separation of the OFT is not taking place, although NCCs have been reported to migrate to this area.^96^, ^99^, ^100^, ^103^ The situation in reptiles varies, as only in crocodilians and birds is OFT separation complete, whereas in lizards, snakes, and turtles separation is more akin to that of amphibians. This does not imply that the OFT septal complex in crocodilians, birds, and mammals is identical (Figure 4). We have to realize that separation in crocodilians results in the formation of three arterial trunks: a pulmonary trunk (connected to the sixth pharyngeal arch arteries) a visceral and a systemic aorta (left and right fourth pharyngeal arch arteries), but in birds and mammals only one aorta persists as outflow of the heart. In birds, the early embryonic left visceral aortic arch artery will regress from the OFT at a time slightly before the completion of ventricular septation, while the right one will persist as the definitive aorta. In mammals, the left aortic arch artery will develop into the definitive aorta, while the right‐sided arch artery will become incorporated into the subclavian artery. This implies that in these different taxa, the cellular partners in the OFT septal complex will show different spatial patterns.^104^ The OFT septal complex includes NCCs and SHF mesenchymal cells.^104^, ^153^, ^154^ Furthermore, condensed mesenchymal prongs suggestive of NCCs^155^, ^156^ co‐populate two OFT endocardial cushions, the septal cushion (dorsally located) and the parietal (ventral) one. The function of these prongs, originally hypothesized to be involved in OFT septation, is unclear, as these are present in both unseptated (turtles) and septated (crocodilians) reptiles, as well as in birds and mammals. After normal remodeling of the human OFT, the septal complex is incorporated into cardiac structures, such as the subpulmonary infundibulum, and cannot be recognized anymore as a septal structure.^157^ Abnormal remodeling leading to congenital OFT malformations, however, may show persisting remnants of the septal complex.^122^ Recently it was reported that in a common arterial trunk (CAT) with an unseptated OFT, NCC‐derived prongs were present in the endocardial cushions. The NCCs, hampered by abnormal SHF positioning with diminutive pulmonary push, take an aberrant course to the endocardial cushions.^154^

The toolboxes govern mechanisms such as endoderm signaling, EMT in the endocardium, mesenchymal‐epithelial transition in the SHF, proliferation to increase the size of the involved cell populations, recruitment of NCCs, migration of SHF and NCCs, interactions between SHF and NCCs, and the differentiation to their final phenotypes, even including apoptosis of NCCs.^92^ The sum of these interacting mechanisms leads to remodeling of the OFT. It comes as no surprise that improper OFT remodeling accounts for a majority of congenital cardiovascular malformations in newborns, whereas an unknown number of affected embryos could have been lost in pregnancy.

## 11 THE ATRIAL SEGMENT

**Figure 6:**
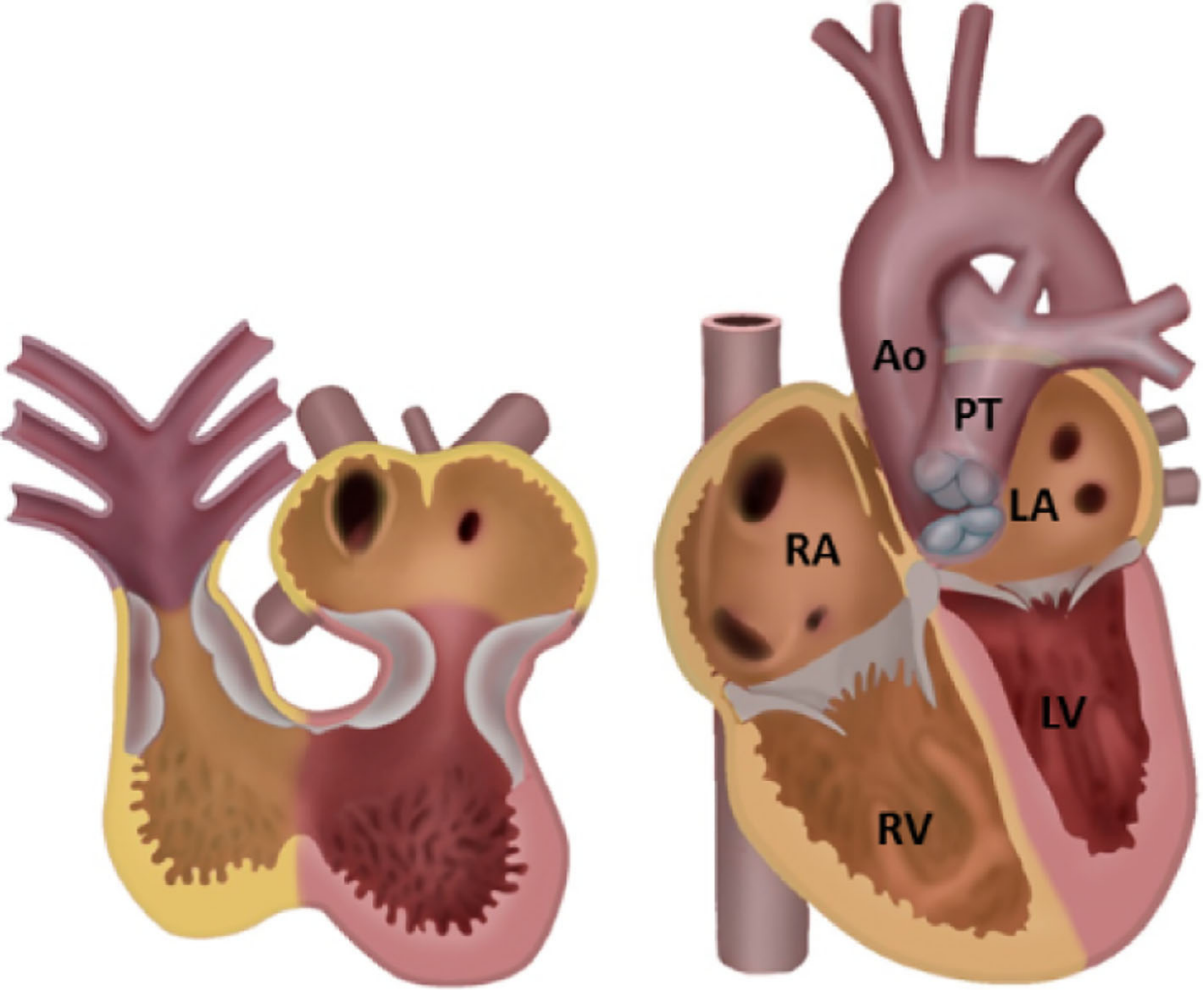
Two stages in cardiac development viewed from ventral. The endocardial OFT and AV cushions involved in septation are depicted in grey. The SHF‐derived regions are in yellow. Left: Atrium and ventricle are not septated yet. Right: Four‐chambered heart. The left ventricle is FHF‐derived; the main components of the other chambers are probably SHF‐derived. Note that the posterior walls of both right and left atria are SHF‐derived, as this represents the incorporated sinus venosus including, for the left atrium, the pulmonary vein myocardium. Ao, Aorta; FHF, first heart field; LA, left atrium; LV, left ventricle; OFT, outflow tract; PT pulmonary trunk; RA, right atrium; RV right ventricle; SHF, second heart field (Adapted from Gittenberger‐de Groot et al^232^)

In vertebrates, a considerable remodeling of the inflow segment of the heart is taking place due to the advent of air‐breathing and the consequence of pulmonary venous return to be separated from the systemic circulation. In other words, the single systemic entry will be joined by the pulmonary entry, realizing a double inlet. In early vertebrate development, the sinus venosus segment of the primary heart tube will feed the (common) atrium (Figure 6). As the sinus venosus is myocardialized in many species, it can be considered a true cardiac chamber. It will remain recognizable as a separate structure in hagfish connected to the left side of the common atrium,^41^ but in birds and most mammals (except monotremes^158^) it will become incorporated in the dorsal atrial wall^159^ (Figure 6). In lungfish, a separate pulmonary channel traverses the main atrial compartment and delivers oxygenated blood directly to the ventricle.^87^ The posterior SHF gives rise to additional cells, leading to expansion of the appendages^160^ of the atrium. The mechanism by which SHF‐derived cells become inserted into the cardiac tube has not been analyzed in detail. Tbx5 mutation as in Holt‐Oram syndrome is characterized by atrial septal defects and occasional right lung agenesis^161^ apart from upper limb malformation. Evolutionary loss of lungs in nearly two‐thirds of the salamanders that, as a consequence, have become obligatory skin‐ and buccopharyngeal‐breathers has a dramatic impact on the configuration of the circulatory system, particularly on cardiac morphology. In these species, the atrium is only partially septated. In amphibians, the lungs induce atrial septation similar to mammals, and atrial septum reduction results directly from reduced or absent lungs.^162^ As these species lack pulmonary veins, all the blood flowing into the heart derives from the cardinal veins via the sinus venosus in the right‐sided part of the atrium, while pulmonary ostia in the left atrium are lacking. During normal development, the pulmonary veins become incorporated into the left atrial body wall (frog^163^) but not the atrial appendage (human^164^), demonstrating the close relationship between the left atrium and lung circulation.

The atrial myocardium derives from the posterior SHF (reviewed in Carmona et al^165^) in both Zebrafish^47^ and the mouse embryo.^76^ In the latter, left/right identity is governed by Pitx2c expression patterns,^166^, ^167^ which is also reported in the agnathan lamprey.^168^ The lineage of the venous pole has been established in podoplanin‐mutant mice^159^ by Tbx18 expression^75^ and by clonal analysis.^53^ Caval vein myocardium has a distinct origin from the SHF based on its transcriptional profile,^75^ sometimes called the tertiary heart field.^169^ In early development, the pulmonary veins are connected to the sinus venosus segment in the left atrium.^170^ There is a common SHF contribution to the venous pole,^159^ segregating into left and right components, with no indication of an independent lineage for caval vein myocardium.^53^ Unexpectedly, a lineage relationship was also found between the venous pole and part of the arterial pole, which is derived exclusively from the SHF.^53^ Note that the left superior caval vein in human will be transformed into the coronary sinus. In the human population, deficient PDGF signaling is related to pulmonary vein abnormalities.^171^ A major structure in atrium development found in many amphibians and in amniotes (reptiles, birds, mammals) is the atrium septum,^172^ dividing the common atrium into left‐ and right‐sided chambers. The primary foramen is closed collaboratively by the AV cushions, the mesenchymal cap, and the DMP, tissues that can also be found in lungfish.^172^ In placental mammals, septation is completed by addition of the second septum, while definitive closure of the septum is completed only after birth.

## 12 THE CARDIAC CONDUCTION SYSTEM

The *Drosophila* heart is composed of an open tube consisting of an anterior aorta and a posterior heart containing ostia drawing hemolymph.^173^ A caudally located autonomic‐acting myogenic pacemaker is present, containing an ensemble of ion‐channels from which the majority is also found in humans.^174^ Neuroregulators also found in vertebrates, such as acetylcholine, serotonin, and norepinephrine, modulate heart rate in larval, pupal, and adult flies.^175^, ^176^ In cephalopods, the systemic heart and the two branchial hearts are similarly under neuroregulatory control,^177^ suggestive of common regulatory pathways. The activity of the heart in other mollusks strongly depends on the function of surrounding organs such as the gut and the excretory system, as well as on muscle activities related to burrowing movements, for example.^178^

**Figure 7:**
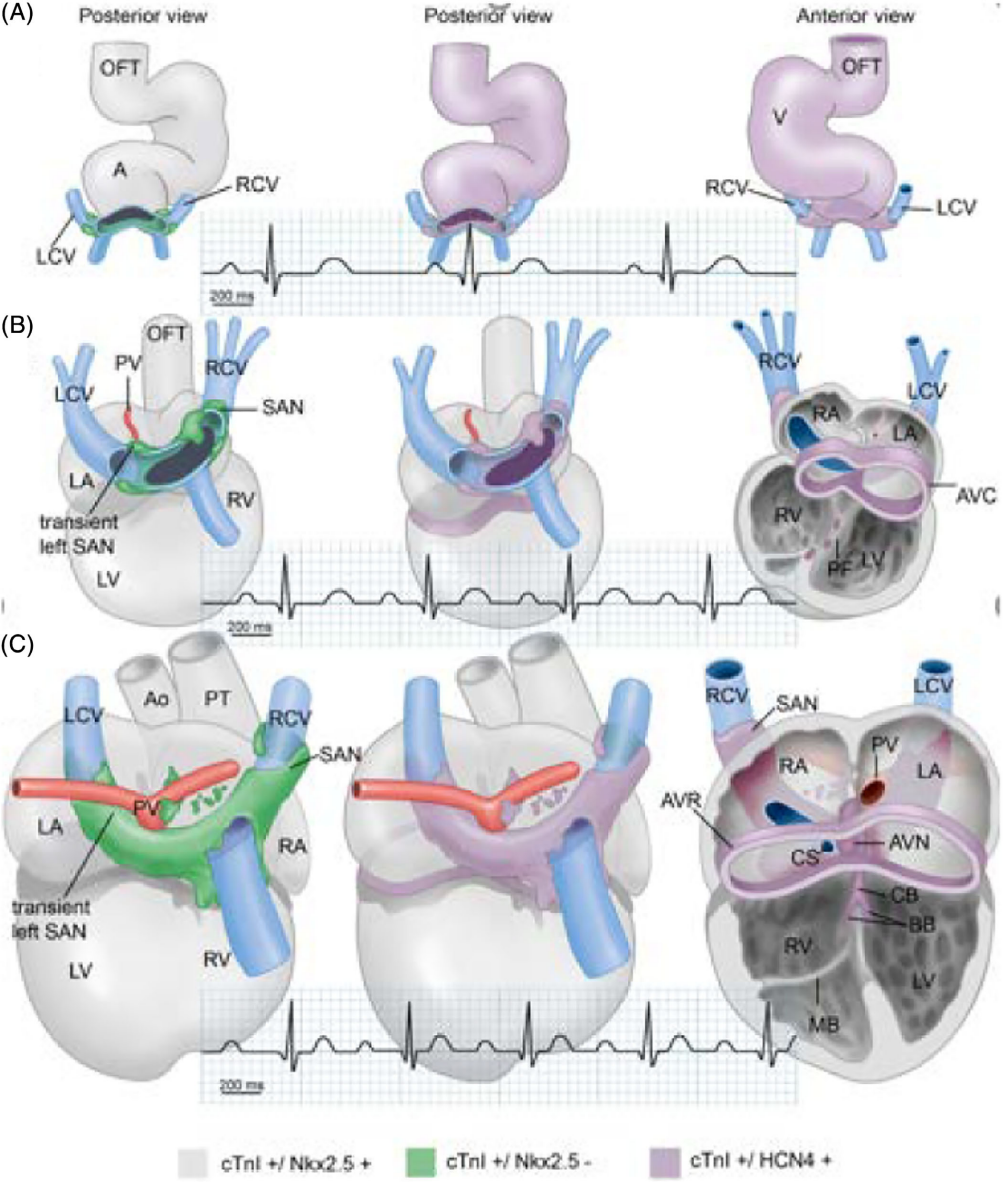
Developing sinus venosus, cardinal veins (blue), and atrium showing the flux of expression of cTnI, Nkx2.5, and HCN4. The pulmonary veins are in red. The definitive right‐sided sinoatrial node and the transient left‐sided sinoatrial node are indicated. Note the lack of Nkx2.5 expression in the developing SANs and the myocardium surrounding the sinus venosus before complete incorporation. There is also an increasing association of HCN4 expression with the conduction system. The cardiogram shows the increasing cardiac frequency during development. SAN, sinoatrial node (from Vicente Steijn et al^180^)

**Figure 8:**
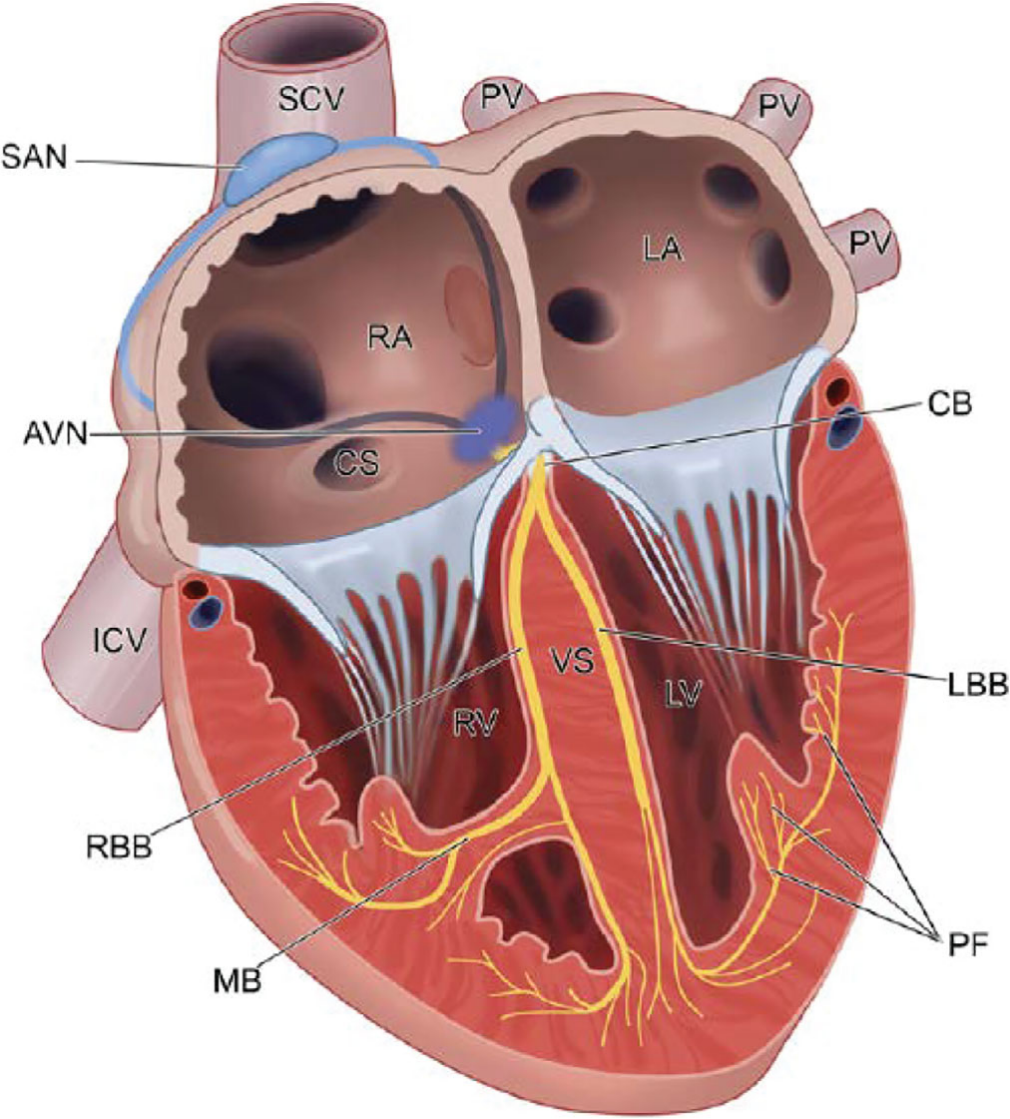
The adult cardiac conduction system. The electrical impulse is generated in the SAN at the entrance of the superior caval vein into the right atrium. It is conducted through the internodal atrial myocardium to the atrioventricular node, where it is delayed. The impulse is then propagated through the common bundle or His bundle and the left and right bundle branches to the Purkinje fiber network. AVN, atrioventricular node; CB, common bundle; CS, coronary sinus, IVC, inferior vena cava; LBB, left bundle branch; LV, left ventricle; MB: moderator band; PF, Purkinje fiber network; PV, pulmonary vein, RA, right atrium; RBB, right bundle branch; RV, right ventricle; SAN, sinoatrial node; SCV, superior caval vein (From Jongbloed et al^233^)

In vertebrates, pacemaker function is a prerequisite for coordinated atrioventricular contraction of the heart and to maintain its rhythmic activity (Figure 7). The cardiomyocytes are electrically coupled, and a highly synchronized activity is governed by the cardiac pacemaker^179^ located at the inflow of the heart in the wall of the sinus venosus that is, in origin, a central compartment feeding the atrium (Figure 7). During lateralization, pacemaker activity will become both left‐ and right‐sided phenomena, eventually restricted to the right side to become the definitive sinus node.^180^ The pacemaker has connections to the atrial cardiomyocytes,^181^ probably in the form of preferential internodal pathways,^182^, ^183^, ^184^, ^185^ as confirmed with electrophysiology,^186^ connecting the sinus node to the AV node (Figure 8).

In early development, peristaltic movements push the blood toward the arterial pole, but slightly later fast propagation of the depolarizing impulse results in synchronous contraction.^115^ During differentiation of the AV canal separating the atrial from the ventricular cardiomyocytes, the AV node develops and delays the contraction of the ventricle. In mammals, it shows a distinct morphology,^187^ but its localization in chicken is still obscure.^188^ Insulation of atrial from ventricular myocardium is a prerequisite for proper conduction via the common bundle. For effective insulation, epicardium‐derived mesenchymal cells (EPDCs) migrate between atrium and ventricular myocardium,^129^ leaving only the AV node as gateway. In case of failing insulation, accessory pathways may persist with ensuing reentrant arrhythmias.^189^

A specialized central AV conduction system comparable to that of birds and mammals is probably not present in fish, amphibians, or reptiles. Among others, the transcriptional repressor Tbx3 is required for the development of the interventricular septum, as well for as the central conduction system.^190^ Crocodilians, the only reptiles with a fully septated four‐chambered heart, seem to occupy an intermediate position, but an SA node and AV node are reported to be absent.^191^ Homologues of mammalian conduction system markers have been identified together with a functional AV bundle.^192^ The peripheral ventricular Purkinje system, however, is absent, and ventricular conduction relies on the trabeculated myocardium, comparable to other ectotherm vertebrates and as also found in early embryonic bird and mammalian hearts. Jensen et al^192^ suggested that the development of the ventricular Purkinje system is strongly associated with high metabolism and endothermy, involving high heart rates. However, this provides on a partial explanation, as early avian and mammalian embryos exhibit high heart rates and temperatures, while many reptilian eggs are incubated at relatively elevated temperatures. It is interesting to note that a strong left‐right asymmetry of the bundle branches develops^193^ together with both ventricular walls and the interventricular system, where the derivatives of the FHF and SHF join back‐to‐back.^79^, ^80^, ^104^

A peripheral Purkinje system is reported in both birds^194^, ^195^ and mammals^196^ and must have developed independently during evolution of ventricular septation and endothermy. The remaining questions here are how the peripheral conduction systems have developed in these taxa and how the connections with the central conduction axis have been made.^197^

## 13 CARDIAC DEVELOPMENT AND THE ACQUISITION OF ENDOTHERMY IN AMNIOTES

An endotherm is an organism that can maintain its resting body temperature across a range of environmental temperatures for extended periods, whereas ectotherms usually conform to the environmental temperature.^198^ It is evident that endothermy is not restricted to birds and mammals, as several insects (scarab beetles, flying insects) and marine fish (tuna), and in some cases turtles, show signs of elevated body temperature for shorter or longer periods of time. These are caused by muscle or metabolic activities and are excluded from this article. Arthropods and mollusks are considered ectotherms. Chordate endotherms are restricted to birds and mammals (Figure 1). Nevertheless, there is no sharp or definitive separation between the nature of ectotherms and endotherms, and the term mesotherms has even been introduced to embrace an “in‐between” group of animals.^199^ Mammalian endotherms evolved around 250 million years ago,^200^ whereas avian endotherms evolved much later, around 65 million years ago.^201^ Not all mammals are considered endotherms. *Echidna* (monotremes) must probably be considered mesotherm with an elevated body temperature only during egg incubation.^202^

In endotherm amniotes (mammals and birds), there is a connection between cardiac output, separation of low pulmonary and high systemic pressure by complete cardiac septation, and high rates of metabolism, which are often 5 to 10 times as high as in reptiles of the same size. Ectotherms usually exhibit a low metabolism combined with a cardiovascular system that lacks complete separation of systemic and pulmonary circulations. The faster heart chamber activation in endotherms, a dominant factor for cardiac output, cannot be explained solely by temperature difference. The compact myocardial architecture in birds and mammals facilitates conduction, resulting in shortening of the cardiac cycle^197^ and increasing the cardiac output. Quantifying time shifting or heterochrony^203^ in evolutionary patterns showed that cardiac development is closely linked to the increasing demands of the terrestrial “closed” amniote egg,^204^ with an independent role for angiogenesis,^205^ both important factors determining respiration and food uptake already in the egg. The ensuing adaptations in the cardiovascular anatomy and function made possible the advent of endothermy.^204^

However, one group of ectotherms with a (near) circulatory separation exists as an exception: crocodilians. Here, the heart is functionally fully septated, although two left/right shunts exist already in the embryo: the central foramen of Panizza and the peripheral shunt between the two aortas.^104^, ^114^, ^206^, ^207^ Seymour et al^206^ argued that crocodilian ancestors were endotherms based on adult characteristics such as anatomy and behavior. We agree with Seymour et al,^206^ postulating that, as a consequence, these terrestrial ancestors would already have realized a septated ventricle, effecting separation of high‐ and low‐pressure circulation. When in the ancestral line and how specific the steps in realization is a matter of speculation. The current special position of the ectothermic extant crocodilians may then be explained by the loss of endothermy during evolution related to a change in activity from land‐dwelling to aquatic ambush behavior.

Arguments based on the characteristics of the cardiovascular architecture as early as in the egg likely precede other theories about the evolutionary onset of endothermy, such as change in juvenile and adult growth parameters,^208^ adult metabolic rate,^199^, ^209^ or oxygen handling and mitochondrial activity.^210^ A further argument from embryo‐related studies came from analysis of fossil egg shells. The eggshell of Titanosaurid fossils (belonging to a sister group of the crocodilians) has been examined for isotopic minerals, reconstructing the body temperature of egg‐laying females.^201^ This temperature proved to be similar to that of large modern endotherms. Ancestral egg shells from Oviraptoridae (closely related to modern birds), on the other hand, demonstrated only a slightly elevated body temperature (mesothermy), suggesting that endothermy has been acquired later in birds.^201^ The fossil record of embryonic and juvenile stages is poor; therefore, the introduction of amniote embryonic cardiovascular physiology as done here can be made only by comparative approaches.

## 14 EVO‐DEVO ASPECTS OF CONGENITAL MALFORMATIONS

Congenital heart disease (CHD) is a major manifestation of incorrect signaling during embryonic development and is conservatively estimated to amount to about 5% of registered births in the western world.^1^ The prenatal demise is unknown, but in a series of early studies encompassing knockouts in mice, an estimated 50% of prenatal lethality is due to malfunctioning of the cardiovascular and/or hemopoietic system.^6^ It is evident that the myriad phenotypes of congenital human malformations must not be considered as interrupted or persisting steps in the evolution of the heart, but rather as results of mutations, imbalance in dosage, or “mismanagement” in the available (genetic) tool kits, including epigenetic regulation,^211^ hemodynamics,^67^, ^212^ and external factors such as vitamin A/retinoic acid.^56^, ^213^

**Figure 9:**
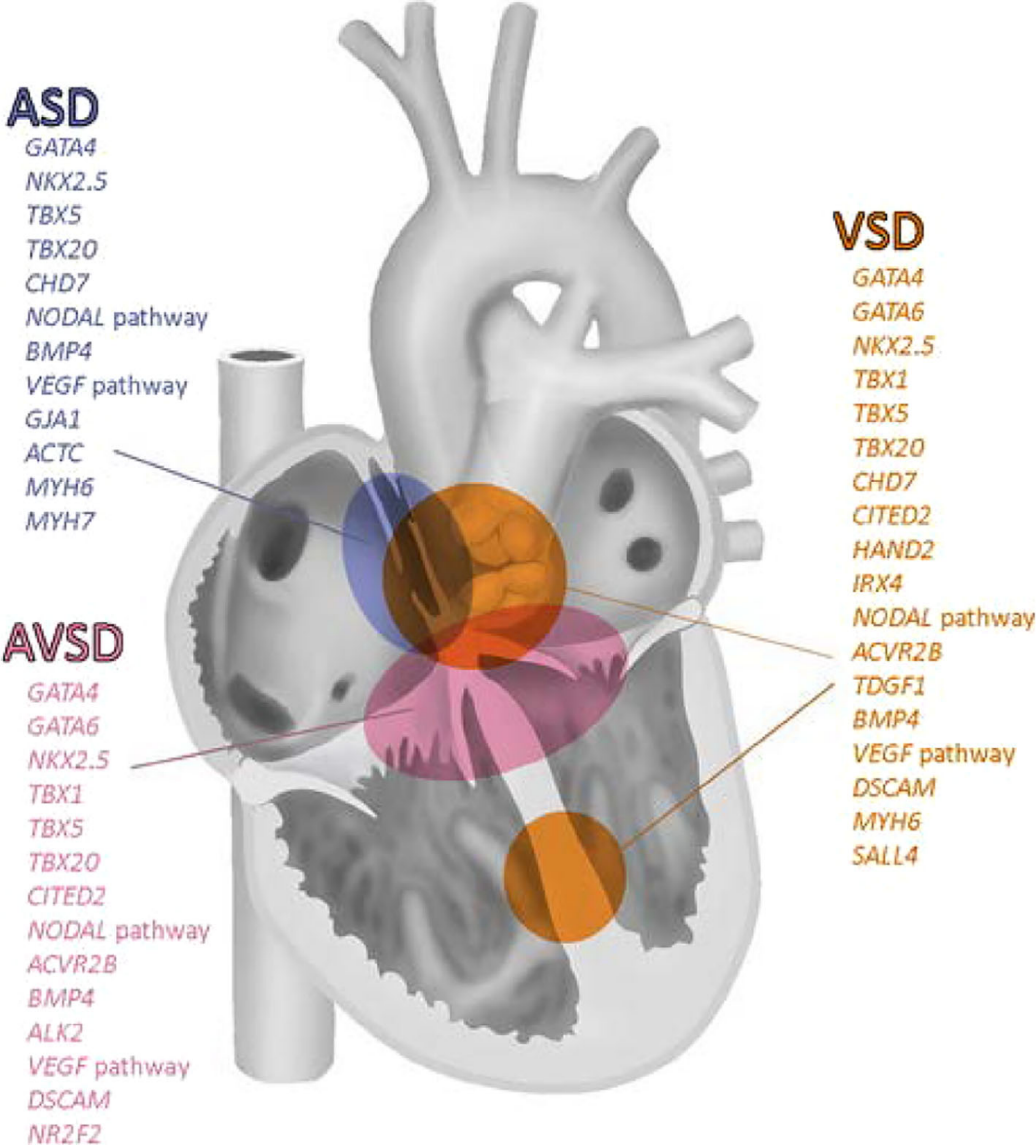
Gene mutations in ASD, AVSD and VSD. The same genes are often involved in the formation of multiple segments of the heart, whereas a given segment might be governed by many genes. ASD, atrial septal defect; AVSD, atrioventricular septal defect: VSD, ventricular septal defect (from Gittenberger‐de Groot et al^108^)

The complexity of interactions leading to CHD may be inferred from large cohort studies in which mutations of many genes^214^ and parts of epigenetic pathways^66^ are involved. The diverse outcomes prevent pinpointing major gene regulatory networks, as is strengthened by the survey of a large cohort description showing that a mere 11% out of more than 9700 patients with CHD (69% of which had “conotruncal” or ventricular OFT defects) had a genetic diagnosis.^214^ These encompass often complex syndromes, such as 22q11, Kabuki, Alagille, Holt‐Oram, but also de novo mutations affecting multiple organs. Regarding cardiac defects, many genes are related to VSDs and OFT malformations such as Tetralogy of Fallot (TOF), double‐outlet right ventricle (DORV), transposition of the great arteries (TGA), and CAT. Similar malformations in animal models have been described above, and include GATA4/6, Nkx2.5, Tbx1/5/20, Hand2, Nodal pathway, BMP2/4, VEGF pathway, and others. Most of these are also involved in atrial septal defects (ASDs) and atrioventricular septal defects (AVSDs) (Figure 9; reviewed in Gittenberger‐de Groot et al^108^).

As reported, we do not consider a congenital malformation as resembling a persistent situation of a normal morphology encountered in fish or reptilian hearts, for example. Most malformations in the human population appear to be specifically linked to the differentiation into a four‐chambered heart and can be spontaneous or experimentally evoked in animal models like the often used (transgenic) mouse or even in the four‐chambered chicken heart. We propose that deviations in the employment of tool kits can be explanatory in this respect.

### 14.1 Malformations at the inflow tract of the heart

In all species described with a functional lung circulation, we notice the incorporation of the sinus venosus into the atrial segment of the heart together with the formation of the primary atrial septum. At its base is the DMP, and at the free rim of the septum an endocardial‐derived mesenchymal cap.^215^, ^216^ When the mesenchymal cap fuses with the AV cushions, separation of the right (or tricuspid in mammals) and the left (or mitral) orifices takes place. In mammals, a malformation may occur in the form of a primary atrial septal defect (ASD type I), or in a more serious case, an AVSD. It has been observed that the DMP is not as developed and the mesenchymal cap is very diminutive.^216^, ^217^, ^218^, ^219^ Genes involved in the tool kit acting in normal and, when corrupt, in abnormal development include Tbx5,^72^ PDGFRα,^171^, ^218^ and Sonic hedgehog.^216^ It is interesting to note that disturbance of podoplanin can also lead to abnormal pulmonary vein connections, which normally are guided to the left atrium by the DMP.^159^ As non‐crocodilian reptiles do not possess an interventricular septum, the occurrence of a congenital AVSD is here impossible.

As the atrium in the four‐chambered heart should remain patent to allow oxygenated blood to reach the left side, the atrial septum must not close completely. Here, reptiles, birds, and nonplacental mammals present secondary perforations in the primary atrial septum, which obliterate after birth/hatching by myocardial and endocardial overgrowth. Placental mammals (Eutheria) display a different approach. The opening in the primary atrial septum that allows right‐left shunting closes after birth by fusion with a secondary atrial septum because of increased pressure in the left atrium. The secondary septum is a right‐sided in‐folding of the atrial wall that partly overlaps the opening in the primary septum. Abnormalities in size of both primary and secondary atrial septal components can lead to a persistent foramen ovale or, in more serious deficiencies, to an ASD type II. Failure to differentiate may lead to AVSDs.^73^, ^219^ Molecular mechanisms controlling atrium and DMP development include PDGF signaling,^171^, ^218^ BMP, Tbx5, Osr1, and FoxF1.^220^, ^221^ Tissue‐specific deletion of Smoothened resulted in compromised DMP formation, AVDs, reduced proliferation, and diminished Wnt/β‐catenin signaling.^216^ In humans, ASDs account for about 9% of more 9700 patients, which is far less than the 69% of children with OFT malformations.^214^ This difference may be a reflection of the difference in complexity in evolution and development of the venous and arterial poles of the heart.

### 14.2 Ventricular septal defects

Complete ventricular septation resulting in a four‐chambered heart has developed separately in mammals and birds but, as we will argue, not completely in crocodilians. Ventricular septal components are found in the inlet of the heart as a diminutive structure consisting of trabecular muscles in, for example, snakes and turtles, found on the posterior wall of the ventricular chamber. The terminology varies and could be of positional (vertical) or more functional (inlet) nature. In complete septation, an anteriorly located folding (horizontal) septum is positioned between the inflow tract (IFT) and OFT of the ventricle.^80^ The final component in closure in the four‐chambered heart consists of the OFT septal complex. This structure contains both specific SHF and NC‐derived cells. The genetic tool kit contains Tbx1, FGF8/10, and TGFβ, among others. We have recently described how in mammals, birds, and reptiles, the contribution of SHF and NC cells varies, resulting in a vascular OFT and two or three main arteries. In lung‐breathing species, this always consists of pulmonary and systemic circulation.

Important in complete septation is the role of endocardial cushions in both the OFT and AV canals. They glue together the various septal components. It is relevant to realize that secondary myocardialization of the ensuing mesenchymal septal components is variable across species. So the OFT septal complex in crocodilians separates the aortic and pulmonary trunks and remains mesenchymal, while in mammals, the muscular right ventricular OFT results from myocardialization guided by TGFβ.^222^, ^223^ At the crossroads of atrial and ventricular septation, usually a membranous septum of variable size is found. VSDs are usually encountered at areas where septal components fuse with more or less mesenchymal boundaries, resulting in clinically relevant classifications such as mesenchymal or perimembranous VSD.^108^, ^224^

Here, we need to discuss the enigmatic complete ventricular septation in crocodilians, which are supposedly secondarily developing an opening between the right aorta (leaving the left ventricle) and left aorta (departing from the right ventricle), the so‐called foramen of Panizza.^114^, ^207^ It turns out that the membranous ventricular septum is extremely large and nonmuscularized.^191^ The foramen of Panizza develops as a tunnel inside one of the endocardial OFT cushions^104^ that is proximal to the actual vessel wall in the mesenchymal area of the semilunar valves. This foramen must, therefore, be considered as a specific shunting communication at the ventricular level, refuting the idea of complete ventricular septation in crocodilians.

### 14.3 Bicuspid semilunar valves

We all have been educated by the elegant and seminal work of Leonardo Da Vinci, suggesting that a semilunar valve carrying three cusps is more efficient compared to a bicuspid valve. However, in the human a bicuspid valve is the most common congenital heart “malformation,” often going undetected until later in adult life and then usually associated with aortic aneurysm.^143^ Recent investigations using transgenic mice demonstrated that mutated “OFT genes” (NOTCH, eNOS, GATA4/5, NKx2.5, and TGFβ pathway, compiled by DeWaard and Postma^225^) are associated with bicuspidy, most likely by disturbed SHF addition and neural crest disturbances. Considering reptiles, we know that these animals usually have bicuspid valves in their three OFT vessels and seem not to be encumbered by this situation.

In both mammals and birds, but not in crocodilians, a solution for the addition of the third cusp is found by adding an intercalated swelling.^133^, ^226^, ^227^ All these authors agree on the importance of the SHF but vary in their explanation for the development of bicuspidy by describing either a non‐anlage of intercalated cusps or a non‐disjunction from the main endocardial OFT cushion.^133^ The difference in incidence of bicuspidy in the aortic vs the pulmonary orifice might have a hemodynamic background related to the difference in mass addition of SHF cells between the aortic and pulmonary sides (as might be inferred from Scherptong et al^89^). The employment of the tool kit genes guiding SHF and NCC addition can lead to abnormal development of the semilunar valves. Interference with these genes and constituents may lead to malformed valves and leaflets, such as in bicuspid aortic valve, often associated with aortic coarctation^228^ and increased susceptibility for aortic aneurysm,^229^ and linked to conditions such as Turner,^228^ Kabuki,^230^ and Marfan syndromes.^231^

A final consideration on developmental differences and similarities concerns the septation of the OFT and the IFT. In both situations, we see involvement of either anterior or posterior SHF. In the IFT of reptilian species, including birds, the atrial primary septum combines with a mesenchymal DMP. SHF plays a major role, while NCCs are less dominantly present in this region. In the OFT of reptiles, the role of SHF is essential in separating the arterial trunks, whereas the NC is important for the separation of the systemic and pulmonary flows. It is intriguing that, in mammals, a folding principle is found both in the IFT and OFT. At the IFT we see development of the secondary atrial septum by in‐folding of the atrial wall, while at the OFT the pulmonary infundibulum and the ventriculo‐infundibular folds likewise derive from an in‐folding. In mammals, this is most likely an adaptation to stabilize the four‐chambered heart structure.

## 15 CONCLUSIONS

The cardiac regulatory tool kit contains many modulating factors such as epigenetic, genetic, viral, hemodynamic, and environmental, as well as transcriptional activators and repressors, duplicated genes, redundancies, and dose dependencies.

Common gene regulatory networks in heart‐bearing species, including arthropods, mollusks, and chordates, involve Wnt/β catenin, Mesp, and BMP during early determination, followed by Nkx2.5, Tbx5, GATA4, HAND, Shh, retinoic acid signaling, and a limited set of others.

Numerous, sometimes non‐organ‐specific tool kits regulate the components of regional mechanisms such as cell‐cell interactions, EMT, mitosis, cell migration, differentiation, and left−/right‐sidedness that are involved in the development and function of, for example, endocardial cushions, looping, septum complexes, pharyngeal arch arteries, chamber and valve formation, and conduction system. As these tool kits regulate specific mechanisms, the ontogeny of complex syndromes involving cardiac malformations can be explained.

Evolutionary development of the cardiovascular system involved in the demands of the yolk sac circulation likely preceded the advent of endothermy in amniotes. This occurred several times in evolution, first in mammals (maybe independently in two groups of mammals^200^) and later again in birds (and maybe separate in ancestral crocodilians^201^).

Parallel evolutionary traits regulate the development of beating pumps in various taxa, developing in the mesoblast often in conjunction with the gut, lungs, and excretory organs that provide various sets of signaling cues during cardiac development.

It is fascinating to learn that a properly functioning heart is essential from the start of embryogenesis, but most other organs are less so. Via the egg yolk or the womb, food is provided by the yolk sac circulation or via the mother through the umbilical circulation, so the alimentary canal becomes functional for embryo survival only much later, even after hatching or birth. Gas exchange is regulated by the air chamber in egg‐laying amniotes and through the placenta in mammals, so yolk/allantois/amnion and umbilical circulation provide for this. There is definitively a priority for cardiogenesis.

## 16 FUTURE DIRECTIONS/UNSOLVED ISSUES

- The beginning of atrium septation in the amniote heart is seemingly closely linked with the evolution of air‐breathing in vertebrates. The studies of the sequence of events in lungless salamanders are interesting and could be intensified with the search in function of the DMP.
- The basket of pharyngeal arch arteries presents various solutions of transporting the blood from the heart toward different regions of the body. Although many descriptions and analyses have been provided, such as the role of Hox genes, a comprehensive molecular analysis combined with hemodynamic forces is still lacking.
- The role of advancing cardiac development and increased output as a result from the separated systemic and pulmonary blood flows should be more directly related to the evolutionary development of endothermy. As endothermy evolved several times wide apart in paleontological history, this presents multiple entrees for research.
- Mammalian history is particularly interesting, as development of embryos relying on a placenta probably originated after the onset of endothermy. It is intriguing to note that the mesotherm egg‐laying *Echidna* maintains a constant body temperature during egg incubation,^202^ which is favorable for development of the embryo in the pouch. Investigations on the evolution of monotremes related to cardiac development could provide more insight in the origin of endothermy in mammals.
